# Survey of Animal Neoplastic Cases Diagnosed in Nigerian Veterinary Teaching Hospitals, 2000–2017

**DOI:** 10.3390/vetsci11040175

**Published:** 2024-04-13

**Authors:** Iniobong Chukwuebuka Ugochukwu, Iasmina Luca, Amienwanlen Eugene Odigie, Emmanuel Okechukwu Njoga, Nuhu Abdulazeez Sani, James Samson Enam, Wafa Rhimi, Sa’idu Tanko Muhammad, Abdussamad Abubakar, Aliyu Mohammed Wakawa, Patricia Otuh, Taiwo Adebiyi, Onyeka Chidiebere Nwufoh, Ikechukwu Udeani, Tosin Oyeleye, Theophilus Aghogho Jarikre, Sheriff Yusuf Idris, Abdulaziz Bada, Zaid Shehu, Ajadi Tola, Chidi Okonkwo, Chioma Frances Egwuogu, Uchechukwu Nnanna Njoku, Ohiemi Benjamin Ocheja, Joel Dzongor, Barka Grema, Najume Dogowar G. Ibrahim, Celestine O. I. Njoku, Anthony Kojo B. Sackey, Benjamin O. Emikpe, Adamu Yunusa, John Ikechukwu Ihedioha, Balarabe Magaji Jahun, Sunday O. Udegbunam, Shodeinde Vincent O. Shoyinka

**Affiliations:** 1 Department of Veterinary Pathology, University of Nigeria, Nsukka 410001, Nigeria; iniobong.ugochukwu@unn.edu.ng (I.C.U.); john.ihedioha@unn.edu.ng (J.I.I.); shodeinde.shoyinka@unn.edu.ng (S.V.O.S.); 2 Dipartimento di Medicina Veterinaria, Universita degli Studi di Bari, 70010 Bari, Italy; eugene.odigie@yahoo.com (A.E.O.); wafarhimi04@yahoo.com (W.R.); 3 Department of Pathological Anatomy and Forensic Medicine, Faculty of Veterinary Medicine, University of Life Sciences “King Mihai I”, 300645 Timisoara, Romania; 4 Department of Veterinary Public Health and Preventive Medicine, University of Benin, Benin City 300238, Nigeria; 5 Department of Veterinary Public Health and Preventive Medicine, University of Nigeria, Nsukka 410001, Nigeria; njoga.emmanuel@unn.edu.ng; 6 Department of Veterinary Pathology, University of Abuja, Gwagwalada Federal Capital Territory, Abuja 900105, Nigeria; nuhu.sani@uniabuja.edu.ng; 7 Department of Veterinary Pathology, Ahmadu Bello University, Zaria 810107, Nigeria; samsonjamesenam@gmail.com (J.S.E.); sidris@tuskegee.edu (S.Y.I.); ndgibrahim@abu.edu.ng (N.D.G.I.); coi_njoku@yahoo.com (C.O.I.N.); 8 Zaria Veterinary Teaching Hospital, Ahmadu Bello University, Zaria 810107, Nigeria; tmsaidu@abu.edu.ng (S.T.M.); abdulvet.vet171@gmail.com (A.A.); aabada@abu.edu.ng (A.B.); 9 Department of Veterinary Medicine, Ahmadu Bello University, Zaria 810107, Nigeria; drsaniwakawa@yahoo.com (A.M.W.); akbsackey@abu.edu.ng (A.K.B.S.); bjahun@gmail.com (B.M.J.); 10 Department of Public Health and Preventive Medicine, Michael Okpara University of Agriculture, Umudike 440101, Nigeria; otuh.patricia@mouau.edu.ng; 11 Veterinary Teaching Hospital, University of Ibadan, Ibadan 200005, Nigeria; drtkemi@gmail.com; 12 Federal College of Animal Health and Production Technology, Ibadan 200273, Nigeria; onyekanwufoh@gmail.com; 13 Veterinary Teaching Hospital, University of Nigeria, Nsukka 410001, Nigeria; ikechukwu.udeani@unn.edu.ng (I.U.); tosin.kolade@unn.edu.ng (T.O.); sunday.udegbunam@unn.edu.ng (S.O.U.); 14 Department of Veterinary Pathology, University of Ibadan, Ibadan 200005, Nigeria; get2theo@yahoo.com (T.A.J.); banabis2001@yahoo.com (B.O.E.); 15 Usmanu Dan Fodio University Veterinary Teaching Hospital, Sokoto 840101, Nigeria; shehu.zaid@udusok.edu.ng (Z.S.); yunusaadamu2000@yahoo.com (A.Y.); 16 Department of Veterinary Surgery and Theriogenology, Federal University of Agriculture, Abeokuta 111101, Nigeria; ade-vsr@hotmail.com; 17 Department of Veterinary Medicine, Michael Okpara University of Agriculture, Umudike 440101, Nigeria; chidi707@yahoo.com; 18 Veterinary Teaching Hospita, Michael Okpara University of Agriculture, Umudike 440101, Nigeria; frajaks2@yahoo.com; 19 Department of Veterinary Surgery, Michael Okpara University of Agriculture, Umudike 440101, Nigeria; njoku.uchenjoku@gmail.com; 20 Department of Veterinary Physiology and Biochemistry, University of Benin, Benin City 300238, Nigeria; ohiemi.ocheja@uniben.edu; 21 Makurdi Veterinary Teaching Hospital, Joseph Tarka University of Agriculture, Makurdi 970212, Nigeria; dzongor.joel@uam.edu.ng (J.D.); drgrema77gmail.com@yahoo.com (B.G.)

**Keywords:** tumours, neoplasms, benign, malignant, domestic animals

## Abstract

**Simple Summary:**

Neoplasm registries are not a common feature of veterinary oncology practice in Africa. Therefore, this study was conducted by obtaining data for specific breeds, age groups or gender with regards to neoplasm occurrence in Nigeria for 17 years (2000–2017). The institutions included in the study were Ahmadu Bello University, Zaria; Federal University of Agriculture Abeokuta; Federal University of Agriculture, Makurdi; Michael Okpara University of Agriculture, Umudike; University of Abuja; University of Ibadan; University of Nigeria, Nsukka; and Usmanu Dan Fodio University, Sokoto. The highest prevalence was reported in the avian species, with Marek’s disease the most prevalently diagnosed neoplastic disease. Females were more affected compared to males, and tumours from the digestive location prevailed. Little emphasis is given to the appropriate diagnosis and recording of neoplastic cases, therefore, an estimation of the neoplastic cases noted in VTHs in Nigeria was done. In addition to the use of mapping tools, the distribution and the prevalence of specific neoplasms, in different geographical zones of Nigeria, were presented. This study will be beneficial to veterinary clinicians, pathologists and epidemiologists and could form a foundation for subsequent work in veterinary oncology and epidemiology in Nigeria and Africa.

**Abstract:**

Incidence data from 17-year veterinary neoplasm surveillance and registration were reviewed. Most of the neoplastic cases diagnosed in Nigerian veterinary teaching hospitals (VTHs) were in the avian (49%) and canine species (44%). Fewer cases were recorded in the equine (3.2%), bovine (2.4%), ovine (1.5%), caprine (0.3%) and porcine (0.15%) species. Marek’s disease was the most prevalently diagnosed neoplastic disease of domestic animals in Nigerian VTHs from 2000–2017. Also, the Nigerian local breed had a higher mean distribution than any other dog breed and this was statistically significant (*p* < 0.05). Nearly all of the neoplastic cases diagnosed, were found in females (60.4%) and so the mean distribution of sex was statistically significant (*p* < 0.05). The digestive system, with 296 (46.25%) cases, was the anatomic location where the majority of the neoplastic cases were found. However, the mean distribution of different neoplastic anatomic sites was not statistically significant (*p* > 0.05). In conclusion, little emphasis is given to the appropriate diagnosis and recording of neoplastic cases that are diagnosed. The study provides information regarding the prevalence and distribution of tumours in different animal species consulted in Nigeria veterinary teaching hospitals. To illustrate all of this, ArcGIS software was used. Veterinary clinicians, pathologists and epidemiologists from Nigeria may benefit from the results of this study by freely accessing some specific data regarding the breed, the age group or the gender of some animal species diagnosed with different tumours.

## 1. Introduction

The role of livestock in human development is enormous and livestock production is an instrument for socio-economic changes because this leads to improved income and quality of life [1]. The economic importance may include the provision of food, mainly protein, employment, manure, clothing leather, pleasure, protection and income [2,3]. 

Animal production, however, is faced with problems such as diseases like neoplasms, inadequate nutrition, the poor genetic potential of the local stock, marketing, social factors and structural constraints [4]. These are a major threat to the sustainable animal production [5]. Companion animal ownership has experienced an upsurge in economic value, and owners are prepared to spend more on their pets, and companion animals are seen as an “important part of the households” [6,7,8]. All animals are susceptible to developing neoplasms, which can occur across a broad range of species or be species-specific. The frequent tumours of animals include equine sarcoid, benign and malignant melanomas, lymphomas, papillomas, equine granulosa cell tumour, squamous cell carcinomas, equine pituitary adenoma of pars intermedia, rabbit uterine adenocarcinoma, equine pedunculated mesenteric lipomas, transmissible venereal tumour of dogs and Marek’s disease of chickens [9,10,11]. In addition, in modern companion and production animal clinical practice, there is a constant demand for reliable and up-to-date information about the diagnosis, therapy and prevention of diseases [12,13]. Despite this fact, accurate epidemiological veterinary tumour data in Nigeria and Africa at large may still be lacking, while the increasing prevalence of tumours in pet animals can be attributed to various factors, one of which is the trend of animals living longer lives [14]. Neoplasm registries have been established and developed for human medicine since the 1940s, providing valuable insights into cancer epidemiology and trends. In contrast, veterinary neoplasm registries have been relatively fewer in number, are often short-lived, and are sporadic in nature although these surveys and studies based on extensive data collections in veterinary oncology research have covered broader fields, thus contributing to our understanding of cancer in animals [15]. In Nigeria, there is no comprehensive nationwide neoplasm registry for domestic animals, hence the need to conduct this study.

## 2. Materials and Methods

### 2.1. Areas Included in Survey

**Figure 1:**
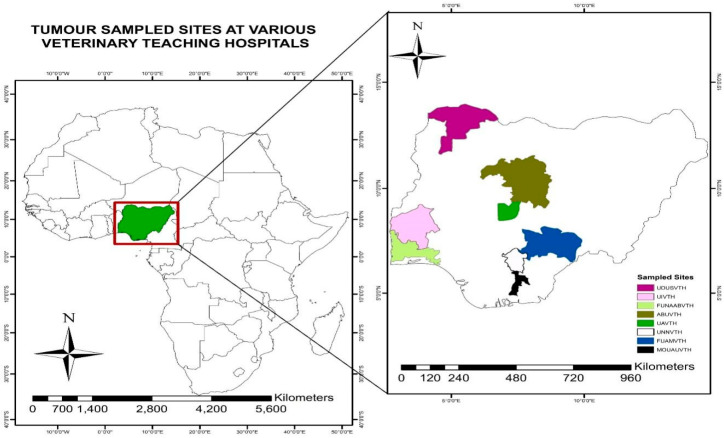
Thematic map showing the country and the localities where the university clinics are located.

This study was conducted through personal visits made at eight VTHs in Nigeria, namely Ahmadu Bello University, Zaria; Federal University of Agriculture Abeokuta; Federal University of Agriculture, Makurdi; Michael Okpara University of Agriculture, Umudike; University of Abuja; University of Ibadan; University of Nigeria, Nsukka; and Usmanu Dan Fodio University, Sokoto (Figure 1) [16].

### 2.2. Study Period 

During the personal visits, the hospital case files from 2000 to 2017 were reviewed and the tumour case profiles were recorded.

### 2.3. Animal Species

The animals included in this study were birds, dogs, cats, horses, pigs, sheep, goats and cattle that presented with neoplasms of any kind. 

### 2.4. Experimental Design 

#### 2.4.1. Sampling Method

VTH individual case files regarding the neoplasms in domestic animals that presented at the selected VTHs nationwide were reviewed. All of the data presenting the medical history and diagnostic information, such as species, sex, breed, age, anatomic site of tumours and gross and histopathologic diagnostic findings, were noted.

#### 2.4.2. Data Presentation and Statistical Analysis 

Data generated from this study were inputted into Microsoft Excel (Microsoft Corporation, Redmond, WA, USA), and exported into R statistical software version 4.0.2.

The distribution of the tumour cases by dichotomous target variables retrieved from the respective VTHs were presented as box plots. The various points on the box plots represent the minimum, first quartile, median, third quartile and maximum values, respectively. Outliers were identified as values greater than twice the product of 1.5 and the interquartile range from the 25 or 75 percentiles.

The spatial (geographical) distribution of neoplasms in the surveyed VTHs and the regions where they are located in Nigeria were analysed using Pearson’s Chi-square test while a one-way analysis of variance (ANOVA) test was computed to test the null hypothesis of no true difference in means of cases and the categorical variables of species and location of sample collection. Additionally, a Tukey post hoc analysis was conducted where a statistically significant difference was obtained at an alpha level of 0.05. 

## 3. Results

### 3.1. Prevalence of Tumours Diagnosed in Domestic Animals in Nigerian VTHs

The prevalence of neoplasms in animals that presented for veterinary care at VTHs, during 2000–2017, in Nigeria, are shown in Table 1. The total number of cases that presented to Nigerian VTHs was 31,500. The most prevalent tumours for each VTH are shown in Table 2. The distribution of benign and malignant tumours among animals are shown in Table 3.

### 3.2. Species Distribution of Domestic Animals that Presented for Veterinary Care at VTHs in Nigeria and were Diagnosed with Tumours, from 2000–2017

**Figure 2:**
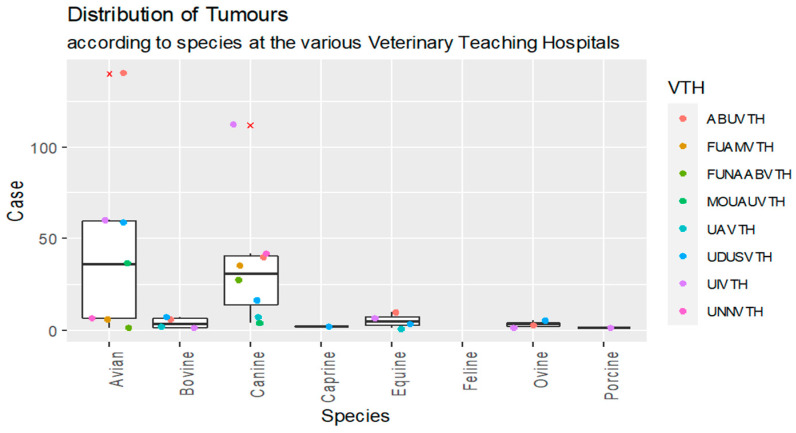
Box plot representation of species distribution of domestic animals that presented for care at veterinary teaching hospitals (VTHs) in Nigeria and were diagnosed with neoplasms from 2000–2017. Note that Case represents number of tumour cases.

The box plot representation of tumours distribution according to species is shown in Figure 2. Of the total cases of tumours, 49% were noted in birds while 44% were encountered in dogs. The other animal species had few cases of neoplasia: 3.2% in horses, 2.4% in cattle, 1.5% in sheep, 0.3% in goats and 0.15% in pigs. The cats consulted in VTHs did not show neoplasia. The geospatial distribution of regions where Nigerian VTHs are located along with the number of the neoplasms diagnosed are shown in Table 4. The Pearson’s Chi-square analysis of geospatial distribution showed that there was a statistically significant difference (0.0006).

**Figure 3:**
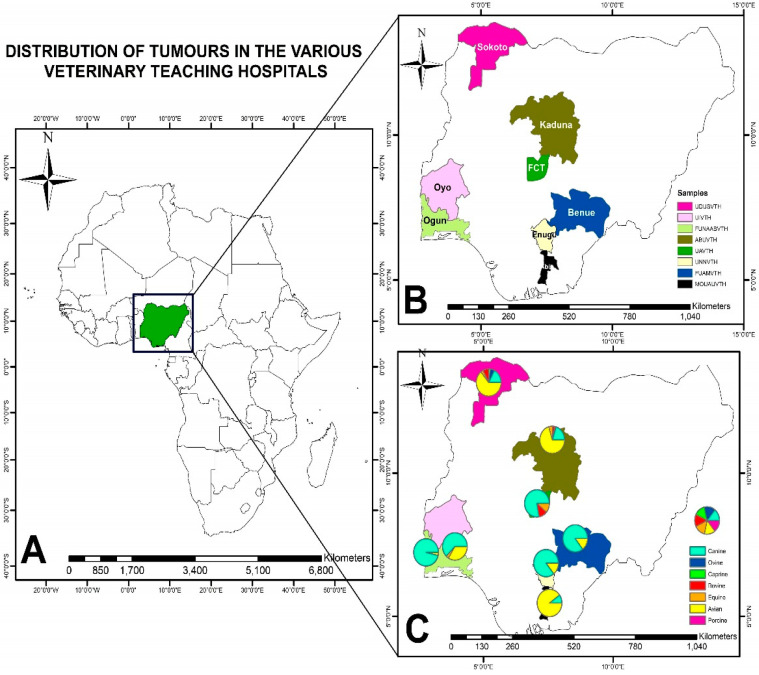
Thematic map showing the study locations for the study (panel (**A**)), and the various sampled locations for neoplasm investigations (panel (**B**)). Panel (**C**) represents the distribution of neoplasms by species in VTHs.

The mean distribution of species diagnosed with neoplasms was not statistically significant (*p* > 0.05). The highest recorded mean distribution was in birds, followed by dogs. There were no significant differences (*p* > 0.05) between VTHs regarding the mean distribution of the total number of tumour cases that were reported. The Arc GIS analysis revealed that most of the neoplasms were in canine species. UDUSVTH and ABUVTH reported cases of tumours in more than half of the investigated domestic animal species (Figure 3).

### 3.3. Breed Distribution of Animals Diagnosed with Neoplasms that Presented for Veterinary Care at VTHs in Nigeria, 2000–2017

**Figure 4:**
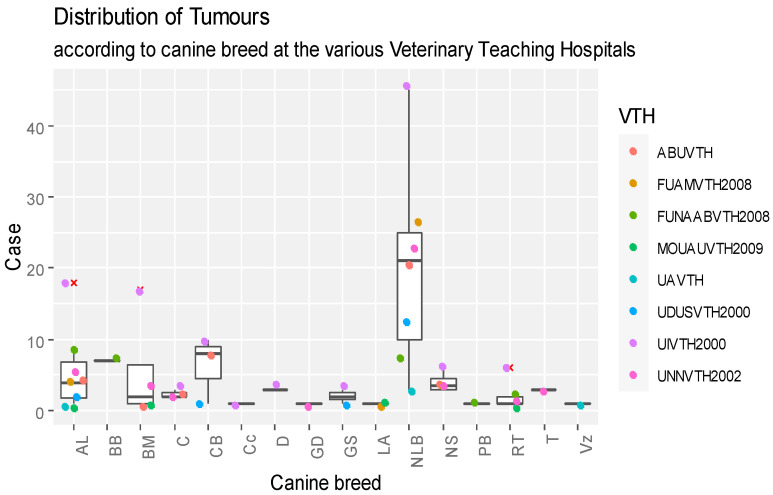
Box plot representation of breed distribution of canines diagnosed with neoplasms that presented for care at VTHs in Nigeria, 2000–2017. Key: GS—German shepherd; NLB—Nigerian local (indigenous) breed; AL—Alsatian; CB—cross breed; LA—Lhasa apso; BB—Boerboel; RT—Rottweiler; PB—Pitbull; T—Terrier; C—Caucasian; GD—Great Dane; D—Doberman pinscher; C_c_—Cane Corso; NS—not specified; Vz—Viszla; BM—Bull Mastiff; ABUVTH—Ahmadu Bello University Veterinary Teaching Hospital, Zaria; FUNAABVTH—Federal University of Agriculture, Abeokuta Veterinary Teaching Hospital, Abeokuta; FUAMVTH—Federal University of Agriculture Makurdi, Veterinary Teaching Hospital, Makurdi; MOUAUVTH—Michael Okpara University of Agriculture Veterinary Teaching Hospital, Umudike; UAVTH—University of Abuja, Veterinary Teaching Hospital, Abuja; UIVTH—University of Ibadan Veterinary Teaching Hospital, Ibadan; UNNVTH—University of Nigeria, Nsukka Veterinary Teaching Hospital, Nsukka; and UDUSVTH—Usmanu Dan Fodio, University Veterinary Teaching Hospital, Sokoto.

Records regarding the dog breeds diagnosed with neoplasms are shown in Figure 4. The Nigerian local breed had a total of 139 tumour cases, more than any other breed of dog. Specifically, as revealed in Figure 4, the highest median distribution value of canine tumour cases was found in NLB breeds, with significant outliers computed for AL, BM, and RT breeds in UIVTH, respectively. Significant differences were recorded between different breeds of dogs diagnosed with tumours (*p* < 0.05). The highest record of the diagnosed tumours in various canine breeds was reported in UIVTH, although the differences between VTHs were not statistically significant (*p* > 0.05). 

**Figure 5:**
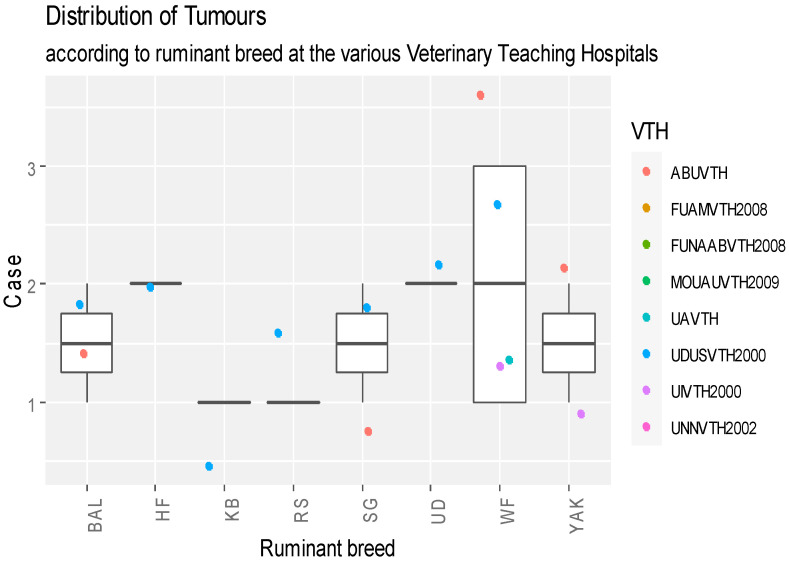
Box plot representation of breed distribution of ruminants diagnosed with neoplasms that presented for care at VTHs in Nigeria, 2000–2017. Key: BAL—Balami; UD—Uda; YAK—Yankasa; RS—Red Sokoto; KB—Kano Brown; WF—White Fulani; HF—Heifer; SG—Sokoto Gudali; ABUVTH—Ahmadu Bello University Veterinary Teaching Hospital, Zaria; FUNAABVTH—Federal University of Agriculture, Abeokuta Veterinary Teaching Hospital, Abeokuta; FUAMVTH—Federal University of Agriculture Makurdi, Veterinary Teaching Hospital, Makurdi; MOUAUVTH—Michael Okpara University of Agriculture Veterinary Teaching Hospital, Umudike; UAVTH—University of Abuja, Veterinary Teaching Hospital, Abuja; UIVTH—University of Ibadan Veterinary Teaching Hospital, Ibadan; UNNVTH—University of Nigeria, Nsukka Veterinary Teaching Hospital, Nsukka; and UDUSVTH—Usmanu Dan Fodio, University Veterinary Teaching Hospital, Sokoto.

Records of the breed distribution of ruminants diagnosed with tumours are shown in Figure 5. The White Fulani breed, having eight neoplastic cases, was the most affected breed of cattle. Regarding the sheep, the Balami breed, with three cases, was the most affected (Figure 5). High records of tumour cases were reported for breeds HF, UD and WF, respectively. Cases prevailed in ABUVTH and UDUSVTH, considering all ruminant species (Figure 5). Even in the case of ruminant breeds, no significant differences were observed between the breeds (*p* > 0.05). The majority of cases were noted in UDUSVTH but no statistically significant differences (*p* > 0.05) were found between the VTHs.

**Figure 6:**
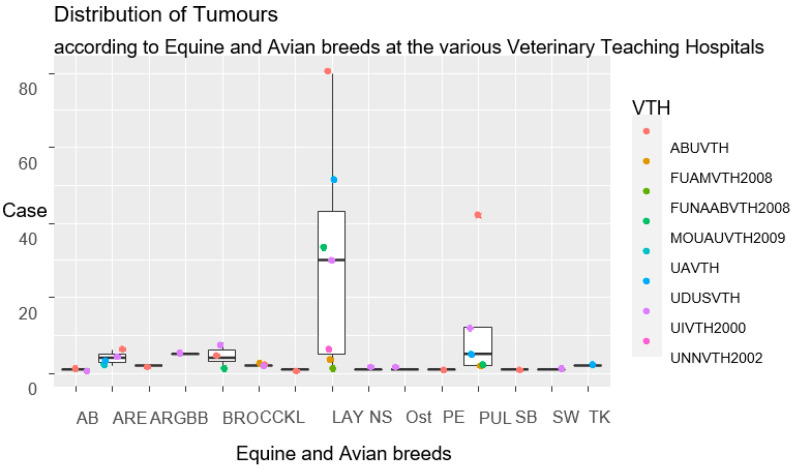
Box plot representation of breed distribution of equines and domestic birds diagnosed with neoplasms that presented for care at VTHs in Nigeria, 2000–2017. Key: ARE—Arewa; SB—Sudan breed; ARG—Argentine breed; AB—Arabian breed; BRO—broilers; CCK—cockerels; LAY—layers; PUL—pullets; BB—broiler breeders; TK—turkey; L—local breed; PE—peacock; Ost—ostrich; SW—swan; NS—not-specified. ABUVTH—Ahmadu Bello University Veterinary Teaching Hospital, Zaria; FUNAABVTH—Federal University of Agriculture, Abeokuta Veterinary Teaching Hospital, Abeokuta; FUAMVTH—Federal University of Agriculture Makurdi, Veterinary Teaching Hospital, Makurdi; MOUAUVTH—Michael Okpara University of Agriculture Veterinary Teaching Hospital, Umudike; UAVTH—University of Abuja, Veterinary Teaching Hospital, Abuja; UIVTH—University of Ibadan Veterinary Teaching Hospital, Ibadan; UNNVTH—University of Nigeria, Nsukka Veterinary Teaching Hospital, Nsukka; and UDUSVTH—Usmanu Dan Fodio, University Veterinary Teaching Hospital, Sokoto.

Records of equine and bird breeds diagnosed with tumours are shown in Figure 6. In the case of horses, the Arewa breed, with 16 cases, was the most affected one. In birds, the layers prevailed, with 211 cases (Figure 6). Therefore, the highest median distribution of cases was observed for layers, although outliers were reported in pullet/chick in ABUVTH (Figure 6).

The differences between the different breeds of equines and birds diagnosed with neoplasia were not statistically significant (*p* > 0.05).

### 3.4. Sex Distribution of Tumours Diagnosed in Domestic Animals in Nigerian VTHs from 2000–2017

Records of the sex distribution of domestic animals diagnosed with neoplasms are shown in Table 5. A total of 134 males and 464 females were diagnosed with neoplasia, while in 41 cases, the sex was not specified. In the case of dogs, 73% (464/639) of the cases were found in females, while 21% (134/639) of cases were found in males.

The mean of the sex distribution of the animals in which the diagnosed neoplasms were observed was statistically significant (*p* < 0.05). The highest mean was in the females while the highest mean of the sex distribution of the animals diagnosed with neoplasms as observed per veterinary teaching hospital showed that UNNVTH had the highest value, although the differences in the means with the other VTHs were not statistically significant (*p* > 0.05).

### 3.5. Frequency of Occurrence of Specific Tumours Diagnosed in Domestic Animals that Presented for Veterinary Care at VTHs in Nigeria, 2000–2017

**Figure 7:**
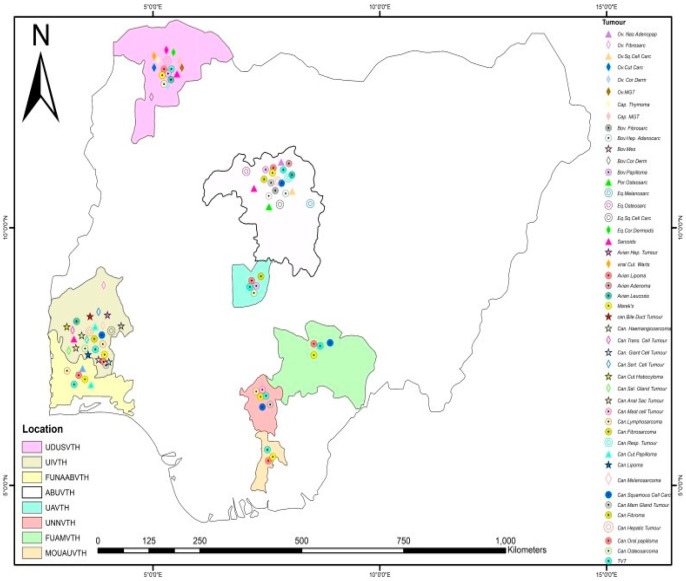
Geographical location of the teaching hospitals and the specific neoplasms diagnosed in each veterinary teaching hospital.

The frequency of the occurrence of specific tumours diagnosed in dogs in Nigerian VTHs are shown in Table 6 while the geographical locations where these tumours were diagnosed are shown in Figure 7. Transmissible venereal tumour, with 153 cases, was the most diagnosed canine tumour. The mean of the distribution of the occurrence of the specific diagnosed canine tumours was not statistically significant (*p* > 0.05). The highest mean was in canine transmissible venereal tumour (CTVT) while the highest record of the total mean of the occurrence of specific diagnosed tumours observed per VTH showed that FUAMVTH had the highest value, although the differences in the means with the other VTHS were not statistically significant (*p* > 0.05).

Records of the frequency of the occurrence of specific neoplasms diagnosed in avian, equine and porcine species in Nigerian VTHs, are shown in Table 7. Marek’s disease, with 281 cases, was the most diagnosed in avian species. Squamous cell carcinoma, with 12 cases, was the most diagnosed in equines while osteoma was the only case of tumour that was diagnosed in porcine species. The mean of the distribution of the occurrence of the specific diagnosed tumours in avian, equine and porcine species was not statistically significant (*p* >0.05). The highest recorded mean was in avian Marek’s disease while the highest record of the mean of the occurrence of specific diagnosed tumours observed per VTH showed that MOUAUVTH had the highest value, although the differences in the means with the other VTHs were not statistically significant (*p* > 0.05).

The frequency of the occurrence of specific tumours diagnosed in ruminants in Nigerian VTHs are shown in Table 8. Bovine papilloma, with seven cases, was the most frequently diagnosed tumour case in ruminants. The mean distribution of the occurrence of the specific diagnosed tumours of ruminants was not statistically significant (*p* > 0.05). The highest mean was in bovine papilloma while the highest record of the mean of the occurrence of specific diagnosed tumours observed in ruminants per VTH showed that UDUSVTH had the highest value, although the differences in the means with the other VTHs were not statistically significant (*p* > 0.05).

### 3.6. Anatomic Locations of Tumours Diagnosed in Domestic Animals that Presented for Veterinary Care at VTHs in Nigeria, 2000–2017

**Figure 8:**
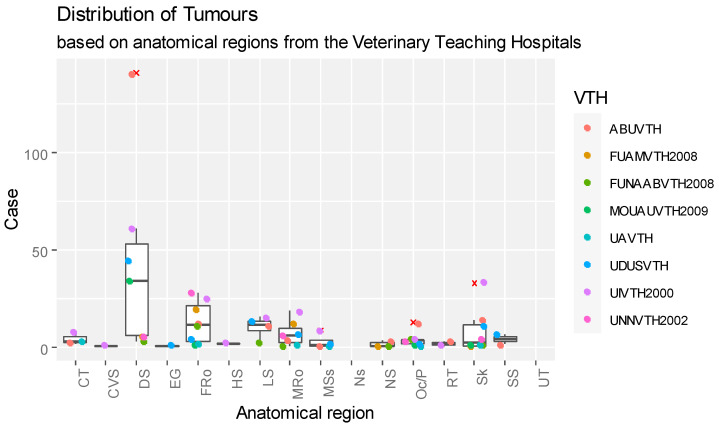
Box plot representation of distribution of diagnosed tumours with respect to anatomic locations of these tumours in domestic animals that presented for care at VTHs in Nigeria, 2000–2017.

The frequency of the occurrence of different diagnosed tumours in different anatomic locations in domestic animals diagnosed with tumours in Nigerian VTHs are shown in Figure 8. The digestive system, with 296 (46.25%) cases, was the location where the tumours were mostly found. Anatomically, the digestive system and the female reproductive system were the body regions mostly affected by tumours with outliers reported in ABUVTH (Figure 8) while ABUVTH and UIVTH also reported outliers for oral cavity/pharynx and skin, respectively.

The mean distribution of the occurrence of the different diagnosed tumours of domestic animals in different anatomic sites was not statistically significant (*p* >0.05). The highest recorded mean was the neoplasms in the digestive system, while the highest recorded mean for the occurrence of diagnosed neoplasms in domestic animals in the different anatomic locations per VTH showed that ABUVTH had the highest value, although the differences in the means with the other VTHs were not statistically significant (*p* > 0.05).

### 3.7. Age Distribution of Domestic Animals Diagnosed with Tumours that Presented for Veterinary Care at VTHs in Nigeria, 2000–2017

The age distribution of canines, bovines, equines, small ruminants (caprines and ovines) and avians with diagnosed tumours in Nigerian VTHs, are shown in Table 9, Table 10, Table 11, Table 12 and Table 13. For the bovines, the yearlings had more tumour cases (four) than any other age group; in canines, equines and small ruminants, the adulthood age group had more cases (127, six and four, respectively) than any other age group, while in avians, the layers age group (217) had more tumour cases than any other age group.

The mean distribution of the occurrence of the different diagnosed canine tumours in different age groups was statistically significant (*p* < 0.05). The highest recorded mean was in the adult age group while the highest recorded total mean of the occurrence of diagnosed canine tumours in different age groups per VTH showed that UIVTH had the highest value and the differences in the means with the other VTHs was also statistically significant (*p* < 0.05). The mean distribution of the occurrence of the different diagnosed tumours for the other domestic animals in different age groups was not statistically significant (*p* > 0.05). The highest mean was recorded in the adult age group for the ruminants, while the highest mean of the occurrence of diagnosed tumours in the different age groups per VTH showed that UDUSVTH and ABUVTH had the highest value for the large and small ruminants, respectively, but the differences in the means with the other VTHs were not statistically significant (*p* > 0.05). 

The mean distribution of the occurrence of the different diagnosed tumours of equines and avians in different age groups was not statistically significant (*p* > 0.05), while the highest mean was recorded in the layers and non-specified age group for horses and avians and the highest recorded mean for the occurrence of diagnosed tumours in different age groups per VTH showed that ABUVTH had the highest value in equines and avians and the differences in the means with the other VTHs were not statistically significant (*p* > 0.05).

## 4. Discussion

Diseases such as neoplasia are a major threat to the sustainable production of high-producing livestock and by extension, a major threat to the health of our pets [5]. 

In this study, most of the diagnosed neoplasms were benign. These findings are different from the findings of Vascellari et al. (2009) [15], who reported equal numbers of malignant and benign neoplasms in dogs. Other researchers have reported more malignant cases than benign ones [17,18].

The identified neoplasms prevailed in bird species, because they outnumber other livestock reared in Nigeria, and it is said that wherever there is human settlement, there are poultry birds [19]. Out of the total number of neoplasms diagnosed in canine species, 49% were observed in the Nigerian local (indigenous) breed. A correlation with these is certain given that this breed of dogs is native in Nigeria. In disagreement with the results obtained are those found by Viana et al. (2019) [17], who reported the Poodle breed as the most affected. Patel et al. (2019) [18], Noury et al. (2020) [18] and Sani et al. (2022) [20], described the German shepherd as the most affected, while Aupperle-Lellbach et al. (2022) [21], reported the Beagles as most affected.

In cattle, almost 75% of the neoplasms were noted in the Bunaji breed, while approximately one fifth of the cases in the bovines were seen in the Sokoto Gudali Breed. This finding might be because the White Fulani (Bunaji) cattle is a widely spread breed in Nigeria. The Bunaji population is closely followed by Sokoto Gudali as the second most popular breed in Nigeria [22]. In ovines, 37.5% of the diagnosed neoplasms were seen in the Balami breed and the Yankasa breed, which are the most common sheep breeds in Nigeria [22]. UDUSVTH from Sokoto had the highest number of neoplastic cases in ruminants (56.5%; 13/23). This could be because the people of Sokoto State, who bring their animals to the UDUSVTH for health care and medical treatment, are mostly pastoralist and these breeds of cattle and sheep are the predominant breeds seen in this part of the country.

In this study, more females were diagnosed with neoplasms than males. This report is similar to the findings of Di Cerbo et al. (2014) [23], Garcia et al. (2019) [24], Noury et al. (2020) [18] and Aupperle-Lellbach et al. (2022) [21]. The results partly disagree with the findings of Sani et al. (2022) [20], who reported most of the neoplasms of dogs in males, while in cats, females were frequently diagnosed. Various researchers have reported skin tumours as the most commonly encountered in practice [17,21,24]. Considering the age of dogs, all individuals under 7 years were the most diagnosed with tumours. The same age group was reported by Viana et al. 2019 [17], Noury et al. 2020 [18] and Sani et al. 2022 [20]. Others reported the senior age group of dogs as being the most affected [21,24]. This finding might be due to the contrast in the level of health care provided to these animals in countries like Mexico, Germany and our study area, Nigeria.

Most of the neoplasms were recorded in older dogs, cattle, birds, sheep, horses and goats. This finding is similar to the findings of Vascellari et al. (2009) [15], who reported similar results in dogs and cats and partly agrees with the findings of Witter et al. (1973) [25], who reported that older birds were fully susceptible to infection with Marek’s disease virus and were only slightly resistant to the development of microscopic lesions. This is also similar to the findings of Sani et al. (2017) [26] for chicken and Khordadmehr et al. (2017) [27], in Pea fowls. Therefore, age, breed and sex are major risk factors for neoplasm development as similarly observed in the reports of Garcia et al. (2019) [24].

The most diagnosed canine neoplasm in Nigerian VTHs was the canine transmissible venereal tumour. This finding differs from the reports of MacVean et al. (1978) [28], Dobson (2013) [29], Aupperle-Lellbach et al. (2022) [21] and Sani et al. (2022) [20]. They mentioned the sebaceous adenoma, the mammary neoplasms and the papilloma as the most common neoplasms found in dogs.

Marek’s disease was the most frequently diagnosed neoplasm in poultry. The same results were reported by Sani et al. (2017) [26], Bertzbach et al. (2020) [30], Nair et al. (2020) [31] and Vychodil et al. (2021) [32]. Avian reticuloendotheliosis was not diagnosed. Sani et al. (2017) [26] reported only Marek’s disease (MD) and avian leukosis (AL) in birds. Cheng et al. (2010) [33] mentioned haemangioma followed by myelocytoma as being the most prevalent. The chicken layers were the most affected by avian neoplasms and this finding is similar to the reports of Cheng et al. (2010) [33], Okonkwo (2015) [34] and Sani et al. (2017) [26]. Dolka et al. (2012) [35] and Othman and Aklilu (2019) [36] recorded more neoplastic disease cases in broilers. In this study, the neoplastic growths were observed in most of the visceral organs in cases of Marek’s disease and lymphoid leukosis. These findings are similar to the reports of Jayalakshmi et al. (2016) [37], Sani et al. (2017) [26] and Stamilla et al. (2020) [38]. The hepatomegaly and splenomegaly seen in cases of avian leukosis and Marek’s disease were also observed by Gopal et al. (2012) [39], Abreu et al. (2016) [40], Othman and Aklilu (2019) [36], Song et al. (2022) [41] and Xu et al. (2022) [42]. 

This study has shown records of tumour cases diagnosed in VTHs in Nigeria for 17 years using Arc GIS to describe the geographical areas where these tumours were diagnosed. A remark from this study is that there is little emphasis given to the appropriate diagnosis and recording of diagnosed tumour cases. It may be a case of nonchalance or complete apathy regarding the subject. In avian oncology, we discovered that in the incidence of a tumour case, after post-mortem diagnosis has been done, the case is recorded as a single one instead of recording the whole number of birds that were brought and which were diagnosed with these neoplastic diseases. Another very important observation was that tumour diagnosis in Nigerian VTHs ends at the histopathology level and in some cases, the diagnosis is made solely based on the observation of gross lesions.

It is important to encourage the proper and continuous documentation of tumour cases. Increasing the database may allow for the evaluation of possible risk factors (species, age, breed, sex, etc.) which could affect the incidence of a particular tumour. Veterinary clinicians, pathologists and especially the oncologists, will benefit from the tumour registry by obtaining data for specific breeds, age groups or gender regarding the tumour occurrence in Nigeria.

## 5. Conclusions

It is recommended that a systematic routine be instituted in veterinary teaching hospitals around the country to document the clinical cases in different animal species, so that every patient’s demographic and medical information can be properly captured. This will be of great assistance to subsequent work and will also help with proper disease surveillance. From the results of this study, it can be seen that incomplete reports and diagnoses were made, therefore it is further recommended that resident doctors and clinicians in VTHs be exposed to regular refresher courses in the area of veterinary oncology and pathology. These will help in proper neoplastic disease diagnosis and curb this apparent apathy towards tumour cases.

## Figures and Tables

**Table 1:** The prevalence of neoplasms in animals that presented for veterinary care at VTHs in Nigeria, 2000–2017.

VTHs in Alphabetical Order	Total Number of Cases that Presented at the VTHs during the Study Period	Total Number of Tumour Cases	% Prevalence of Tumours
Ahmadu Bello University VTH Zaria	11,334	204	1.80%
Federal University of Agriculture Abeokuta VTH, Abeokuta	2293	32	1.40%
Federal University of Agriculture Makurdi VTH, Makurdi	3980	42	1.06%
Michael Okpara University of Agriculture VTH, Umudike	868	39	4.49%
University of Abuja VTH, Abuja	111	9	8.11%
University of Ibadan VTH, Ibadan	7071	182	2.57%
University of Nigeria VTH, Nsukka	3526	49	1.39%
Usmanu Dan Fodio University VTH, Sokoto	2317	92	3.97%
Totals and Overall Prevalence	31,500	649	2.06%

**Table 2:** The most diagnosed neoplasm types in animals that presented for veterinary care at VTHs in Nigeria, 2000–2017.

VTHs in Alphabetical Order	Most Diagnosed Tumour Type for Each VTH
Ahmadu Bello University VTH Zaria	Marek’s disease
Federal University of Agriculture Abeokuta VTH, Abeokuta	Transmissible venereal tumour
Federal University of Agriculture Makurdi VTH, Makurdi	Transmissible venereal tumour
Michael Okpara University of Agriculture VTH, Umudike	Marek’s disease
University of Abuja VTH, Abuja	Transmissible venereal tumour
University of Ibadan VTH, Ibadan	Marek’s disease
University of Nigeria VTH, Nsukka	Transmissible venereal tumour
Usmanu Dan Fodio University VTH, Sokoto	Marek’s disease

**Table 3:** The distribution of benign and malignant tumours among animals that presented for veterinary care in VTHs in Nigeria, 2000–2017.

VTHs in Alphabetical Order	Total Number of Cases that Presented at the VTHs during the Study Period	Total Number of Benign Tumour Cases	Total Number of Malignant Tumour Cases
Ahmadu Bello University VTH Zaria	11,334	190 (1.68%)	14 (0.12%)
Federal University of Agriculture Abeokuta VTH, Abeokuta	2293	27 (1.18%)	5 (0.22%)
Federal University of Agriculture Makurdi VTH, Makurdi	3980	40 (1.01%)	2 (0.05%)
Michael Okpara University of Agriculture VTH, Umudike	868	39 (4.49%)	0 (0%)
University of Abuja VTH, Abuja	111	8 (7.21%)	1 (0.90%)
University of Ibadan VTH, Ibadan	7071	148 (2.09%)	34 (0.48%)
University of Nigeria VTH, Nsukka	3526	42 (1.19%)	7 (0.20%)
Usmanu Dan Fodio University VTH, Sokoto	2317	79 (3.41%)	13 (0.56%)
Totals and Overall Prevalence	31,500	573 (1.82%)	76 (0.24%)

**Table 4:** Geospatial distribution of regions where neoplasms of domestic animals were diagnosed in Nigerian VTHs.

Geopolitical Zones	Total Number Surveyed	Total Number of Cases	Percentage Prevalence	Chi-Square Value	*p*-Value
South-east	4394	88	2%	17	0.0006 *
South-west	9364	214	2.2%		
South-south	-	-	-		
North-east	-	-	-		
North-west	13,651	296	2.5%		
North-central	4091	51	1.2%		

* = Significant *p*-value, Pearson’s Chi-square test.

**Table 5:** Sex distribution of animals diagnosed with neoplasms that presented for care at VTHs in Nigeria, 2000–2017.

Species	Can			Ovi			Cap			Bov			Equ			Avi			Porc		
Univ. VTH/Sex	M	F	NS	M	F	NS	M	F	NS	M	F	NS	M	F	NS	M	F	NS	M	F	NS
ABUVTH ^2000−2017^	10	28	2	-	3	-	-	-	-	2	4	-	5	2	3	3	137	-	-	-	-
FUNAABVTH^2008−2017^	10	17	-	-	-	-	-	-	-	-	-	-	-	-	-	-	1	-	-	-	-
FUAMVTH ^2008−2017^	16	18	1	-	-	-	-	-	-	-	--	-	-	-	-	1	5	-	-	-	-
MOUAUVTH^2009−2017^	2	2	-	-	-	-	-	-	-	-	-	-	-	-	-	-	36	-	-	-	-
UAVTH ^2015−2017^	3	3	1	-	-	-	-	-	-	1	-	-	1	-	-	-	-	-	-	-	-
UIVTH ^2000−2017^	44	41	27	-	-	1	-	-	-	1	-	-	4	1	1	12	48	-	1	-	-
UNNVTH ^2002−2017^	8	34	-	-	-	-	-	-	-	-	-	-	-	-	-	-	7	-	-	-	-
UDUSVTH ^2000−2017^	6	8	2	1	4	-	1	1	-	1	5	1	1	-	2	-	59	-	-	-	-
TOTAL	99	151	33	1	7	1	1	1	-	5	9	1	11	3	6	16	293	0	1	-	-

Key and Totals: Can—canine; Ovi—ovine; Cap—caprine; Bov—bovine; Equ—equine; Avi—avian; Por—porcine; M—males = 134; F—females = 464; NS—non-specific = 41. ABUVTH—Ahmadu Bello University Veterinary Teaching Hospital, Zaria; FUNAABVTH—Federal University of Agriculture, Abeokuta Veterinary Teaching Hospital, Abeokuta; FUAMVTH—Federal University of Agriculture Makurdi, Veterinary Teaching Hospital, Makurdi; MOUAUVTH—Michael Okpara University of Agriculture Veterinary Teaching Hospital, Umudike; UAVTH—University of Abuja, Veterinary Teaching Hospital, Abuja; UIVTH—University of Ibadan Veterinary Teaching Hospital, Ibadan; UNNVTH—University of Nigeria, Nsukka Veterinary Teaching Hospital, Nsukka; and UDUSVTH—Usmanu Dan Fodio, University Veterinary Teaching Hospital, Sokoto.

**Table 6:** Frequency of occurrence of specific neoplasms diagnosed in dogs that presented for care at veterinary teaching hospitals (VTHs) in Nigeria, 2000–2017.

Species	Canine																										
Univ. VTH	TVT	OS	OrP	HA	FB	MGT	SCC	MEL	Lip	CP	RT	FS	LS	L	MC	AST	SGT	CH	ST	P_c_	Lei	NT	LipS	GST	TCT	HS	BDT
ABUVTH^2000−2017^	15	-	12	8	1	2	1	-	-	-	1	-	-	-	-	-	-	-	-	-	-	-	-	-	-	-	-
FUNAABVTH^2008−2017^	18	-	4	-	-	-	-	-	-	1	-	1	2	-	-	-	-	-	-	-	-	-	-	-	-	-	-
FUAMVTH^2008−2017^	30	-	4	-	-	-	1	-	-	-	-	-	-	-	-	-	-	-	-	-	-	-	-	-	-	-	-
MOUAUVTH^2009−2017^	3	-	1	-	-	-	-	-	-	-	-	-	-	-	-	-	-	-	-	-	-	-	-	-	-	-	-
UAVTH^2015−2017^	3	1	1	-	2	-	-	-	-	-	-	-	-	-	-	-	-	-	-	-	-	-	-	-	-	-	-
UIVTH^2000−2017^	40	8	5	4	1	8	4	1	2	1	-	2	6	2	-	3	1	9	6	1	1	1	2	1	1	1	1
UNNVTH^2002−2017^	34	-	1	-	-	4	1	-	-	-	-	-	-	1	1	-	-	-	-	-	-	-	-	-	-	-	-
UDUSVTH^2000−2017^	11	2	2	1	-	-	-	-	-	-	-	-	-	-	-	-	-	-	-	-	-	-	-	-	-	-	-
TOTAL	154	11	30	13	4	14	7	1	2	2	1	3	8	3	1	3	1	9	6	1	1	1	2	1	1	1	4

Key: TVT—transmissible venereal tumour; OS—osteosarcoma; OrP—oral papilloma; HA—hepatic adenocarcinoma; FB—fibroma; MGT—mammary gland neoplasm; SCC—squamous cell carcinoma; MEL—melanosarcoma; LIP—lipoma; CP—cutaneous papilloma; RT—respiratory organ neoplasm; FS—fibrosarcoma; LS—lymphosarcoma; L—lymphoma; MC—mast cell neoplasm; AST—anal sac neoplasm; SGT—salivary gland neoplasm; CH—cutaneous histiocytoma; ST—Sertoli cell neoplasm; P_c_—prostate gland adenocarcinoma; Lei—leiomyosarcoma; NT—nasal neoplasm; LipS—liposarcoma, GST—giant cell neoplasm; TCT—transitional cell neoplasm; HS—hemangiosarcoma; BDT—bile duct neoplasm. ABUVTH—Ahmadu Bello University Veterinary Teaching Hospital, Zaria; FUNAABVTH—Federal University of Agriculture, Abeokuta Veterinary Teaching Hospital, Abeokuta; FUAMVTH—Federal University of Agriculture Makurdi, Veterinary Teaching Hospital, Makurdi; MOUAUVTH—Michael Okpara University of Agriculture Veterinary Teaching Hospital, Umudike; UAVTH—University of Abuja, Veterinary Teaching Hospital, Abuja; UIVTH—University of Ibadan Veterinary Teaching Hospital, Ibadan; UNNVTH—University of Nigeria, Nsukka Veterinary Teaching Hospital, Nsukka and UDUSVTH—Usmanu Dan Fodio, University Veterinary Teaching Hospital, Sokoto.

**Table 7:** Frequency of occurrence of specific neoplasms diagnosed in avian, equine and porcine breeds that presented for care at VTHs in Nigeria, 2000–2017.

Species	Avian						Equine					Porcine
Univ. VTH	Mar	AL	A	L	VCW	HT	Sarc	CD	SCC	OS	MEL	OST
ABUVTH^2000−2017^	127	11	1	1	-	-	2	-	6	1	1	1
FUNAABVTH^2008−2017^	1	-	-	-	-	-	-	-	-	-	-	-
FUAMVTH^2008−2017^	6	-	-	-	-	-	-	-	-	-	-	-
MOUAUVTH^2009−2017^	36	-	-	-	-	-	-	-	-	-	-	-
UAVTH^2015−2017^	-	-	-	-	-	-	-	-	-	-	-	-
UIVTH^2000−2017^	52	6	-	-	-	2	-	-	6	-	-	-
UNNVTH^2002−2017^	7	-	-	-	-	-	-	-	-	-	-	-
UDUSVTH^2000−2017^	44	13	-	-	2	-	2	1	-	-	-	-
TOTAL	273	30	1	1	2	2	4	1	12	1	1	1

Key: Mar—Marek’s; AL—avian leucosis; A—adenoma; L—lipoma; VCW—viral cutaneous warts; HT—hepatic neoplasm; Sarc—sarcoids; CD—corneal dermoids; SCC—squamous cell carcinoma; OS—osteosarcoma; MEL—melanosarcoma; OST—osteoma; ABUVTH—Ahmadu Bello University Veterinary Teaching Hospital, Zaria; FUNAABVTH—Federal University of Agriculture, Abeokuta Veterinary Teaching Hospital, Abeokuta; FUAMVTH—Federal University of Agriculture Makurdi, Veterinary Teaching Hospital, Makurdi; MOUAUVTH—Michael Okpara University of Agriculture Veterinary Teaching Hospital, Umudike; UAVTH—University of Abuja, Veterinary Teaching Hospital, Abuja; UIVTH—University of Ibadan Veterinary Teaching Hospital, Ibadan; UNNVTH—University of Nigeria, Nsukka Veterinary Teaching Hospital, Nsukka; and UDUSVTH—Usmanu Dan Fodio, University Veterinary Teaching Hospital, Sokoto.

**Table 8:** Frequency of occurrence of specific neoplasms diagnosed in ruminants that presented for care at VTHs in Nigeria, 2000–2017.

Species	Bovine					Caprine		Ovine					
Univ. VTH	BP	CD	Mes	HA	FB_s_	MGT	Thy	MGT	CC	CD	SCC	FBS	NA
ABUVTH^2000−2017^	3	-	-	1	2	-	-	-	-	-	1	-	2
FUNAABVTH^2008−2017^	-	-	-	-	-	-	-	-	-	-	-	-	-
FUAMVTH^2008−2017^	-	-	-	-	-	-	-	-	-	-	-	-	-
MOUAUVTH^2009−2017^	-	-	-	-	-	-	-	-	-	-	-	-	-
UAVTH^2015−2017^	1	-	-	-	-	-	-	-	-	-	-	-	-
UIVTH^2000−2017^	-	-	1	-	-	-	-	-	-	-	-	1	-
UNNVTH^2002−2017^	-	-	-	-	-	-	-	-	-	-	-	-	-
UDUSVTH^2000−2017^	4	3	-	-	-	1	1	1	2	2	-	-	-
TOTAL	8	3	1	1	2	1	1	1	2	2	1	1	2

Key: BP—bovine papilloma; CD—corneal dermoids; Mes—mesothelioma; HA—hepatic adenocarcinoma; FB_s_—fibrosarcoma; MGT—mammary gland neoplasms; Thy—thymoma; MGT—mammary gland neoplasm; CC—cutaneous carcinoma; CD—corneal dermoids; SCC—squamous cell carcinoma; FBS—fibrosarcoma; NA—nasal adenopapilloma; ABUVTH—Ahmadu Bello University Veterinary Teaching Hospital, Zaria; FUNAABVTH—Federal University of Agriculture, Abeokuta Veterinary Teaching Hospital, Abeokuta; FUAMVTH—Federal University of Agriculture Makurdi, Veterinary Teaching Hospital, Makurdi; MOUAUVTH—Michael Okpara University of Agriculture Veterinary Teaching Hospital, Umudike; UAVTH—University of Abuja, Veterinary Teaching Hospital, Abuja; UIVTH—University of Ibadan Veterinary Teaching Hospital, Ibadan; UNNVTH—University of Nigeria, Nsukka Veterinary Teaching Hospital, Nsukka; and UDUSVTH—Usmanu Dan Fodio, University Veterinary Teaching Hospital, Sokoto.

**Table 9:** Age distribution of dogs diagnosed with neoplasms that presented for care at veterinary teaching hospitals (VTHs) in Nigeria, 2000–2017.

Univ. VTH/AG	* Day Old–6 mnths Puppyhood	* 6 mnths–1 yr	* 1 yr–5 yrs Adulthood	* 6 yrs–10 yrs Old	* 10 yrs and up Geriatric	NS
ABUVTH^2000−2017^	1	6	15	7	4	7
FUNAABVTH^2008−2017^	1	1	12	2	1	10
FUAMVTH^2008−2017^	1	4	19	6	-	5
MOUAUVTH^2009−2017^	1	2	1	-	-	-
UAVTH^2015−2017^	1	1	3	2	-	-
UIVTH^2000−2017^	3	1	44	33	14	17
UNNVTH^2002−2017^	-	5	27	3	2	5
UDUSVTH^2000−2017^	-	2	6	1	-	7
TOTAL	8	22	127	54	21	51

* Key-Univ. VTH—University Veterinary Teaching Hospital; AG—age group; NS—not specified; mnths—months; yr(s)—year(s); ABUVTH—Ahmadu Bello University Veterinary Teaching Hospital, Zaria; FUNAABVTH—Federal University of Agriculture, Abeokuta Veterinary Teaching Hospital, Abeokuta; FUAMVTH—Federal University of Agriculture Makurdi, Veterinary Teaching Hospital, Makurdi; MOUAUVTH—Michael Okpara University of Agriculture Veterinary Teaching Hospital, Umudike; UAVTH—University of Abuja, Veterinary Teaching Hospital, Abuja; UIVTH—University of Ibadan Veterinary Teaching Hospital, Ibadan; UNNVTH—University of Nigeria, Nsukka Veterinary Teaching Hospital, Nsukka; and UDUSVTH—Usmanu Dan Fodio, University Veterinary Teaching Hospital, Sokoto.

**Table 10:** Age distribution of cattle diagnosed with neoplasms that presented for care at veterinary teaching hospitals (VTHs) in Nigeria, 2000–2017.

Univ. VTH/AG	* Day old–a few mnths Calves	* Few mnths–1 yr Weaners	* 1 yr–2 yrs Yearlings	* 2 yrs–3 yrs Young Ox(M)/Heifer(F)	* 4 yrs and up Adult	NS
ABUVTH^2000−2017^	-	-	2	1	3	-
FUNAABVTH^2008−2017^	-	-	-	-	-	-
FUAMVTH^2008−2017^	-	-	-	-	-	-
MOUAUVTH^2009−2017^	-	-	-	-	-	-
UAVTH^2015−2017^	-	-	1	-	-	-
UIVTH^2000−2017^	-	-	-	-	-	1
UNNVTH^2002−2017^	-	-	-	-	-	-
UDUSVTH^2000−2017^	3	-	1	-	-	3
TOTAL	3	-	4	1	3	4

* Key-Univ. VTH—university veterinary teaching hospital; AG—age group; M—male; F—female; NS—not specified; mnths—months; yr(s)—year(s); ABUVTH—Ahmadu Bello University Veterinary Teaching Hospital, Zaria; FUNAABVTH—Federal University of Agriculture, Abeokuta Veterinary Teaching Hospital, Abeokuta; FUAMVTH—Federal University of Agriculture Makurdi, Veterinary Teaching Hospital, Makurdi; MOUAUVTH—Michael Okpara University of Agriculture Veterinary Teaching Hospital, Umudike; UAVTH—University of Abuja, Veterinary Teaching Hospital, Abuja; UIVTH—University of Ibadan Veterinary Teaching Hospital, Ibadan; UNNVTH—University of Nigeria, Nsukka Veterinary Teaching Hospital, Nsukka; and UDUSVTH—Usmanu Dan Fodio, University Veterinary Teaching Hospital, Sokoto.

**Table 11:** Age distribution of horses diagnosed with neoplasms that presented for care at veterinary teaching hospitals (VTHs) in Nigeria, 2000–2017.

Univ. VTH/AG	* Day old–6 mnths Foals	* 6 mnths–1 yr Colt	* 2 y–15 yrs Adulthood	* 15 yrs and up Old	NS
ABUVTH^2000−2017^	-	-	3	2	5
FUNAABVTH^2008−2017^	-	-	-	-	-
FUAMVTH^2008−2017^	-	-	-	-	-
MOUAUVTH^2009−2017^	-	-	-	-	-
UAVTH^2015−2017^	-	-	-	-	1
UIVTH^2000−2017^	-	-	2	-	4
UNNVTH^2002−2017^	-	-	-	-	-
UDUSVTH^2000−2017^	-	-	1	-	2
TOTAL	-	-	6	2	12

* Key-Univ. VTH—university veterinary teaching hospital; AG—age group; NS—not specified; mnths—months; yr(s)—year(s); ABUVTH—Ahmadu Bello University Veterinary Teaching Hospital, Zaria; FUNAABVTH—Federal University of Agriculture, Abeokuta Veterinary Teaching Hospital, Abeokuta; FUAMVTH—Federal University of Agriculture Makurdi, Veterinary Teaching Hospital, Makurdi; MOUAUVTH—Michael Okpara University of Agriculture Veterinary Teaching Hospital, Umudike; UAVTH—University of Abuja, Veterinary Teaching Hospital, Abuja; UIVTH—University of Ibadan Veterinary Teaching Hospital, Ibadan; UNNVTH—University of Nigeria, Nsukka Veterinary Teaching Hospital, Nsukka; and UDUSVTH—Usmanu Dan Fodio, University Veterinary Teaching Hospital, Sokoto.

**Table 12:** Age distribution of small ruminants diagnosed with neoplasms that presented for care at veterinary teaching hospitals (VTHs) in Nigeria, 2000–2017.

Univ. VTH/AG	* Day old–4 mnths Kid/Lamb	* 1 yr Yearling	* 2 y–7 yrs Adulthood	* 8–15 yrs Old	NS
ABUVTH^2000−2017^	-	-	2^+^	-	1^+^
FUNAABVTH^2008−2017^	-	-	-	-	-
FUAMVTH^2008−2017^	-	-	-	-	-
MOUAUVTH^2009−2017^	-	-	-	-	-
UAVTH ^2015−2017^	-	-	-	-	-
UIVTH^2000−2017^	-	-	1^+^	-	-
UNNVTH^2002−2017^	-	-	-	-	-
UDUSVTH^2000−2017^	1	-	1	-	-
TOTAL	1	-	4	-	1

* Key-Univ. VTH—university veterinary teaching hospital; AG—age group; NS—not specified; mnths—months; yr(s)—year(s); ABUVTH—Ahmadu Bello University Veterinary Teaching Hospital, Zaria; FUNAABVTH—Federal University of Agriculture, Abeokuta Veterinary Teaching Hospital, Abeokuta; FUAMVTH—Federal University of Agriculture Makurdi, Veterinary Teaching Hospital, Makurdi; MOUAUVTH—Michael Okpara University of Agriculture Veterinary Teaching Hospital, Umudike; UAVTH—University of Abuja, Veterinary Teaching Hospital, Abuja; UIVTH—University of Ibadan Veterinary Teaching Hospital, Ibadan; UNNVTH—University of Nigeria, Nsukka Veterinary Teaching Hospital, Nsukka; and UDUSVTH—Usmanu Dan Fodio, University Veterinary Teaching Hospital, Sokoto.

**Table 13:** Age distribution of domestic avian species diagnosed with neoplasms that presented for care at veterinary teaching hospitals (VTHs) in Nigeria, 2000–2017.

Univ. VTH/AG	* Day old–17 wks Pullet/chick	* 18 wks and up Layers	* 12–18 mnths Hen	* 18 mnths and up Molting Chicks	NS
ABUVTH^2000−2017^	42	89	4	-	5
FUNAABVTH^2008−2017^	-	-	1	-	-
FUAMVTH^2008−2017^	2	4	-	-	-
MOUAUVTH^2009−2017^	2	33	-	-	1
UAVTH^2015−2017^	-	-	-	-	-
UIVTH^2000−2017^	14	39	1	2	4
UNNVTH^2002−2017^	-	6	1	-	-
UDUSVTH^2000−2017^	4	46	6	-	3
TOTAL	64	217	13	2	13

* Key-Univ. VTH—university veterinary teaching hospital; AG—age group; NS—not specified; wks—weeks; mnths—months. ABUVTH—Ahmadu Bello University Veterinary Teaching Hospital, Zaria; FUNAABVTH—Federal University of Agriculture, Abeokuta Veterinary Teaching Hospital, Abeokuta; FUAMVTH—Federal University of Agriculture Makurdi, Veterinary Teaching Hospital, Makurdi; MOUAUVTH—Michael Okpara University of Agriculture Veterinary Teaching Hospital, Umudike; UAVTH—University of Abuja, Veterinary Teaching Hospital, Abuja; UIVTH—University of Ibadan Veterinary Teaching Hospital, Ibadan; UNNVTH—University of Nigeria, Nsukka Veterinary Teaching Hospital, Nsukka; and UDUSVTH—Usmanu Dan Fodio, University Veterinary Teaching Hospital, Sokoto.

## Data Availability

Supporting data of this work could be made available upon request from the first Author.
